# Submovement Composition of Head Movement

**DOI:** 10.1371/journal.pone.0047565

**Published:** 2012-11-05

**Authors:** Lewis L. Chen, Daeyeol Lee, Kikuro Fukushima, Junko Fukushima

**Affiliations:** 1 Department of Otolaryngology, Neurobiology and Anatomical Sciences, Ophthalmology, University of Mississippi Medical Center, Jackson, Mississippi, United States of America; 2 Department of Neurobiology, Yale University Medical School, New Haven, Connecticut, United States of America; 3 Department of Physiology, Hokkaido University School of Medicine, Sapporo, Japan; 4 Department of Health Science, Hokkaido University School of Medicine, Sapporo, Japan; University of Regensburg, Germany

## Abstract

Limb movement is smooth and corrections of movement trajectory and amplitude are barely noticeable midflight. This suggests that skeletomuscular motor commands are smooth in transition, such that the rate of change of acceleration (or jerk) is minimized. Here we applied the methodology of minimum-jerk submovement decomposition to a member of the skeletomuscular family, the head movement. We examined the submovement composition of three types of horizontal head movements generated by nonhuman primates: head-alone tracking, head-gaze pursuit, and eye-head combined gaze shifts. The first two types of head movements tracked a moving target, whereas the last type oriented the head with rapid gaze shifts toward a target fixed in space. During head tracking, the head movement was composed of a series of episodes, each consisting of a distinct, bell-shaped velocity profile (submovement) that rarely overlapped with each other. There was no specific magnitude order in the peak velocities of these submovements. In contrast, during eye-head combined gaze shifts, the head movement was often comprised of overlapping submovements, in which the peak velocity of the primary submovement was always higher than that of the subsequent submovement, consistent with the two-component strategy observed in goal-directed limb movements. These results extend the previous submovement composition studies from limb to head movements, suggesting that submovement composition provides a biologically plausible approach to characterizing the head motor recruitment that can vary depending on task demand.

## Introduction

It has been shown that skeletomuscular movements exhibit a smooth bell-shaped velocity profile that could be described as minimizing the rate of change of acceleration, or jerk [1]. This notion has been supported by the observations of different limb movement tasks [2]–[4]. In particular, when movement trajectory and amplitude were modified midflight, movement transition remained smooth. The movement could be described as consisting of multiple submovements, each composed of a bell-shaped velocity profile that overlapped with one another [2]–[8]. These findings were taken to suggest that the generation of limb movements involved overlapping motor commands, and that each of these worked through muscle synergies which were in turn expressed as a minimum-jerk submovement [9], [10]. Whether this applies to head movements has not been demonstrated.

Previous limb movement studies also showed that patients recovering from strokes improved over time, such that their movements consisted of fewer and longer submovements [11]–[13]. That is, the change in submovement composition occurred in parallel with the change in movement proficiency and motor recruitment. This suggests that it is likely that submovement composition reflects movement dynamics. This also implies that motor recruitment varies depending on task demand, and thus is associated with different patterns of submovement configuration [14], [15]. For example, when a limb movement was made to a target fixed in space, initial submovements tended to be larger in amplitude and have a higher peak velocity than subsequent submovements [4], [16]–[18]. This is the so-called “two-component strategy” first proposed by Woodworth (1899). This strategy accounts for the kinematics of limb movements, in which the initial movement tends to be large and is often followed by relatively smaller, corrective movements [17]. In contrast, when a limb movement intercepted or tracked a moving object, there was no systematic magnitude relationship among the submovements [5], [8]. Whether the two- component strategy is present in goal-directed head movements is currently unknown.

The present study addressed the above questions. We examined head movements obtained during three different tasks performed by nonhuman primates. The target was either continuously moving (i.e. head-alone tracking and head-gaze pursuit) or fixed in space (i.e. eye-head combined gaze shifts). Our findings indicate that the minimum-jerk model [1], [5] can adequately describe the submovement composition of head movements in a task-dependent manner.

## Methods

### Ethics Statements, subjects, and recording of gaze and head positions

Five juvenile rhesus monkeys (2 *Macaca fuscata* and 3 *Macaca mulatta*, 4–7 kg) served as subjects. The procedures for surgical implants, coil recording, animal training, and euthanasia were approved by the Animal Care and Use Committee at Hokkaido University School of Medicine at Sapporo, Japan (2 *Macaca fuscata*) [19] and the INSERM U848 Ethics Committee at Lyon, France (3 *Macaca mulatta*) [20], [21]. The findings reported here are limited to the data obtained from these animals. These animals were housed with *ad-lib* food and water, and were attended to by full-time veterinarians. During the experiments, the animals' water intake was rescheduled based on the operant conditioning procedures. Specific procedures that enhanced animals' well-being and minimized distress were described in detail previously and all animals were euthanized with an overdose of pentobarbital [19]–[21]. During testing, the monkeys were seated facing straight ahead in primate chairs. Their torso and shoulder movements were limited using Styrofoam blocks. Conventional search-coil techniques were used to record gaze (eye re space) and head (re space) position signals at 500 Hz. Horizontal eye (re head) positions were computed as the difference of the horizontal gaze and head positions.

### Behavioral procedures

**Figure 1:**
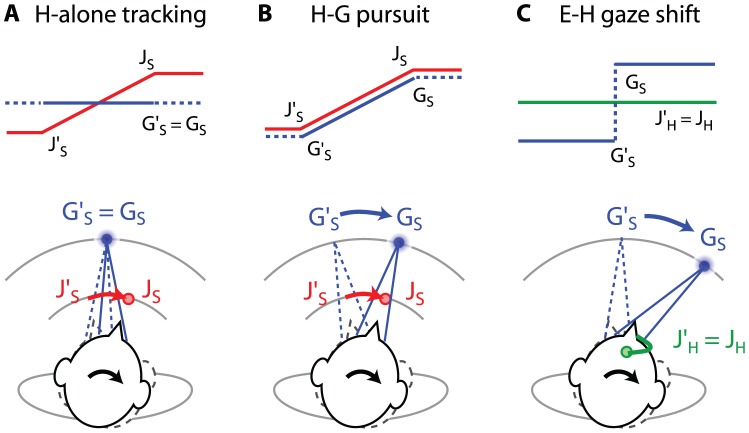
Schematics of target configuration during head-alone (H-alone) tracking, head-gaze (H–G) pursuit and eye-head combined (E–H) gaze shifts. G_S_: gaze target re space (blue). J_S_: juice spout position re space (red). J_H_: juice spout position re head (green). G′_S_, J′_S_, and J′_H_ represent the initial positions for gaze target re space, juice spout re space, and juice spout re head, respectively. Note during head-alone tracking and head-gaze pursuit, the juice spout was rotated in the same angular velocity as the gaze target motion, whereas during eye-head combined gaze shifts, the juice spout was attached to and rotated with the head.

Two animals (M1 and M2) were trained to perform head movement during 2 tasks: head-alone tracking (Fig. 1A) and head-gaze pursuit (Fig. 1B; for details, see [19]). During both tasks, head rotation was limited to the earth vertical axis by a mechanical coupling. The gaze target was displayed as a red dot (0.2° in diameter) on a LCD screen, which was aligned with the straight-ahead direction. During the head-alone tracking task, the gaze target was displayed at a fixed position in space. During the head-gaze pursuit task, the dot was moved horizontally at 20°/sec with a total of 20° displacement from 10° to the left to 10° to the right. To control head movement independently from eye movement, a motorized juice spout was placed ∼2 mm away from the animal's mouth. The spout was rotated at 20°/s with a total of 20° displacement. In short, during the head-alone tracking task, the gaze target was fixed straight ahead in space, and only the juice spout was rotated. The head traced the spout movement. During the head-gaze pursuit task, both gaze target and juice spout were rotated with the same angular velocity and in the same direction. The monkeys were rewarded randomly at a 500–1000-ms interval for maintaining their gaze and head positions within ±1° from their respective target positions. It was our impression that the animal had no problem performing the head tracking movement when both gaze and spout velocities were the same. We observed significant disruption to the animals' performance when the gaze and spout velocities were different.

Three animals (M3–M5) were trained to perform visually guided gaze shifts (Fig. 1C; [20]). The animal's head was entirely unrestrained, and a juice spout was attached to and rotated with the animal's head. The gaze target was an illuminated LED on a spherical dome. The task started with the animal fixating at the center of the screen. As soon as the fixation point was extinguished, an eccentric (±20–40°) horizontal gaze target was illuminated. There were two gaze target configurations. During the stationary target configuration, the gaze target was illuminated continuously until 200 or 300 ms after gaze completion. During the flashed target configuration, the gaze target was illuminated for 50 ms. The reward delivery was contingent upon the animals making successful gaze shifts to a location within ±5° from the target.

### Determination of submovement composition

Submovement composition of horizontal head movement was determined based on the minimum-jerk model [1], [5]. The minimum-jerk model of head velocity (V^J^) for a given submovement was based on the following equation.
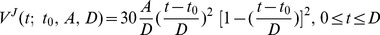
(1)where t_0_ is movement onset, A is movement amplitude, and D is movement duration. That is, the height and width of each velocity template (submovement) was scaled in order to optimize the model fitting. As a result, the linear sum of the submovements was given as follows.
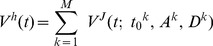
(2)where the observed head velocity (V^h^) is expressed as the sum of the velocities of the submovements (V^J^). M is the number of submovements.

Submovement composition was optimized based on a multidimensional unconstrained nonlinear minimization algorithm in Matlab, “fminsearch” (Mathworks, Co.). Maximal iteration and functional evaluation were set as 200,000. We used the least-squared criterion to minimize the mean squared errors of the model, as follows.
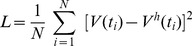
(3)where N is the number of data points (sampled at 500 Hz) between the onset and offset of a given movement. V(t) is the observed head velocity, whereas V^h^(t) is the estimated head velocity (Eq. 2). The goodness of fit, R^2^ value, was provided to indicate the mean squared error between the observed head velocity and the minimum-jerk model.

The algorithm used to find the optimal submovement composition began with a set of the initial parameters described in Eq. 2 [5], [22], [23]. Given the relatively brief task epoch of our study, we opted to estimate these initial parameters visually. Additional sets of parameters, slightly deviating from the initial estimates (±50-ms step in onset, ±2° step in amplitude, and ±50-ms step in duration), were explored to find additional local minima, if any. In <20% of the cases, this search strategy did render more than one configuration of submovement composition, each of which reflected a local minimum with its corresponding mean squared error (Eq. 3). These alternative compositions were carefully examined, and biologically implausible configurations were rejected based on the criteria described below. For automation and effectiveness of global search algorithms, please see Rohrer and Hogan [22], [23].

To avoid including small jitters as movements, the accepted duration of head movement was selected based on velocity thresholds (onset: 15°/s; offset: 25°/s), identified in forward and backward directions. For a given head movement, optimization was first attempted with a single submovement, then additional submovements were added until R^2^ value reached >0.985 (or <1.5% error of the total variance of head velocity). Occasionally (<3% of data), more than one submovement composition exceeded the goodness-of-fit cutoff. In these cases, rejection of implausible configurations resulted in a single, well-defined submovement composition. A typical implausible configuration consisted of an anti-submovement in the inverse direction. The other implausible, non-parsimonious configuration consisted of a submovement overlapping substantially (>60% in duration) with the preceding submovement. This latter case constituted a minority (<1%) of the data, and we opted to select the configuration with one less submovement. This compromised slightly the goodness-of-fit cutoff (R^2^ = 0.965–0.985; see Results) by <1% error.

### Discreteness measures for overlapping submovements

When submovements overlapped one another, the discreteness was quantified by two measures: duration overlap index (DOI) and peak discreteness index (PDI). The DOI was computed as the ratio of the overlapping duration (Od) over the sum of the duration of each submovement (first submovement duration [Fd]+second submovement duration [Sd]). This measure took into consideration only the submovement duration (time) regardless of the amplitude of velocity peaks. The PDI measure quantified how much the first and second submovement peaks were separated from each other. The PDI was computed as the ratio of the velocity peak of the second submovement over the head velocity at the time when the first and second submovements intercepted each other.

## Results

Three types of horizontal head movements were included in the analysis described below: head-alone tracking (M1: N = 43; M2: N = 55), head-gaze pursuit (M1: N = 41; M2: N = 49), and eye-head combined gaze shifts (stationary gaze target: M3: N = 36; M4: N = 90, M5: N = 49; flashed gaze target: M3: N = 48; M4: N = 73, M5: N = 56) (Fig. 1). These head movements were selected only from the task-associated epoch, not the entirety, of the tasks (see Methods for details). Because there was no significant difference in the data across animals, the data was pooled and analyzed based on movement types.

### Submovement composition

**Figure 2:**
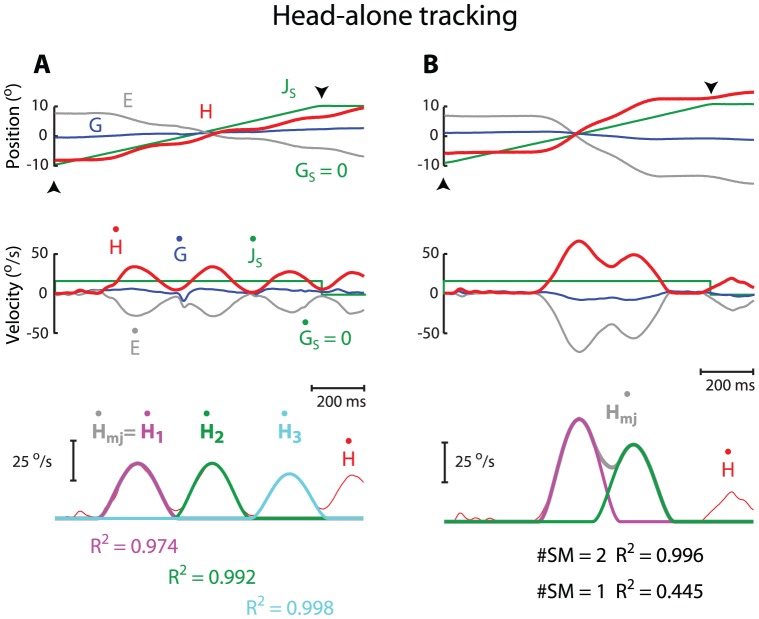
Two examples of submovement composition during head-alone tracking. Top two rows depict position (top) and velocity (middle) traces, respectively, separated for gaze movement (G; blue), head movement (H; red), eye movement (E, re head; gray), and juice spout motion (J_s_; green). Bottom panels depict the profiles of the head velocity (H, thin red) and the minimum-jerk model of head velocity (H_mj_, thick gray). Task-associated head movement was selected between the onset (▴) and offset (▾) of juice spout motion. The order of the submovements is coded in color (first: magenta; second: green; third: light blue). Time scale is identical across all plots. To facilitate data comparison, the head movements are plotted in positive directions regardless of whether the movements were rightward or leftward, the other movements were rectified accordingly. Note the goodness of fit in **B** improved by 55.1% from a single submovement model (#SM = 1; 55.5% error) to two overlapping submovement model (#SM = 2; 0.4% error).

Figure 2 shows two typical examples of submovement composition during head-alone tracking movements. The middle panels illustrate the head velocity (red) profiles of the two head movements. In A, the head velocity increased to its peak and then fell to near zero; this episode repeated 3 times. The 3 discrete velocity episodes corresponded to 3 non-overlapping submovements (Fig. 2A, bottom). In contrast, the head velocity in B increased to its peak, slowed down to ∼30°/s, and climbed again to a second peak before declining to zero. The 2 episodes corresponded to 2 overlapping submovements (Fig. 2B, bottom). Overall, the majority (61%, 60/98) of head-alone tracking movements consisted of non-overlapping submovements, whereas the remaining (39%; 38/98) consisted of 2 submovements that overlapped each other. Head-alone tracking movements with 3 submovements that overlapped one another were never present.

For the case of non-overlapping submovements, the average goodness of fit of the minimum-jerk model was 0.988±0.009 (mean ± S.D.), whereas for the case of overlapping submovements, 0.991±0.008. Note that for the case of non-overlapping submovements, the goodness of fit could be as high as R^2^ = 0.999 (or error of 0.1%), suggesting that the minimum-jerk description was highly consistent and thus a biologically valid characterization of the dynamics of head movement.

**Figure 3:**
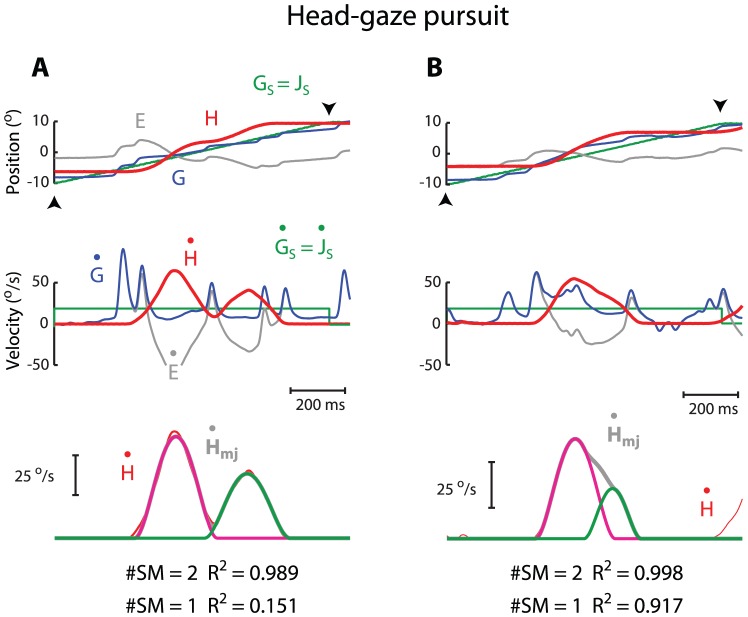
Two examples of submovement composition of head movement during head-gaze pursuit. Note the goodness of fit in **A** improved by 83.8% from one single submovement (#SM = 1; 84.9% error) to 2 overlapping submovements (#SM = 2; 1.1% error). The goodness of fit in **B** improved by 8.1% from a single submovement (#SM = 1; 8.3% error) to 2 overlapping submovements (#SM = 2; 0.2% error). Format after Figure 2.

Figure 3 shows two typical examples of submovement composition during head-gaze pursuit. The presence of eye pursuit combined with head pursuit resulted in a complex pattern of eye movements and associated changes in gaze velocity. However, the head movements seen in this condition were relatively simple. Like head-alone tracking movement, the majority (58%, 52/90) of head-gaze pursuit movements consisted of discrete and non-overlapping submovements. The rest (N = 39) consisted of a maximum of 2 overlapping submovements, e.g., Fig. 3, A and B. For the case of non-overlapping submovements, the average goodness of fit was 0.987±0.009. For the case of overlapping submovements, the average goodness of fit was 0.992±0.009. In other words, imposing gaze pursuit did not appear to alter the submovement composition of the head tracking movement.

**Figure 4:**
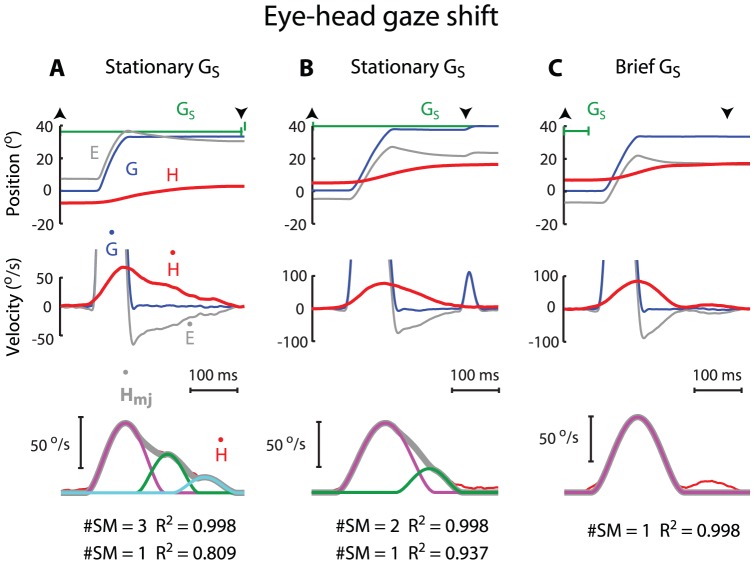
Three examples of submovement composition of head movement during eye-head combined gaze shifts. For stationary gaze target (**A–B**), task-associated head movement, indicated as arrowheads ▴ and ▾, was included up to the end of gaze target display (A) or the onset of correction saccade when correction gaze shifts occurred (B). For flashed gaze target (**C**), task-associated head movement was included up to 200 ms following gaze end. Note the goodness of fit in **A** improved by 18.9% from a single submovement (#SM = 1; 19.1% error) to 3 overlapping submovements (#SM = 3; 0.2% error). The goodness of fit in **B** improved by 6.1% from a single submovement (#SM = 1; 6.3% error) to 2 overlapping submovements (#SM = 2; 0.2% error). Same format as in Fig. 2.

During eye-head combined gaze shifts, the head movement typically showed a rightward-skewed velocity profile (Fig. 4, A and B). When the visual target remained visible during gaze shifts, nearly all (99%, 159/160) head movements consisted of at least 2 overlapping submovements (Fig. 4, A and B). Among these, 79% (N = 126) consisted of 2 overlapping submovements, whereas 21% (N = 33) consisted of 3 overlapping submovements. When the visual target was flashed, 90% (160/177) of the movements included at least 2 submovements; 84% (N = 149) consisted of 2 overlapping submovements, while 6% (N = 11) consisted of 3 overlapping submovements. The rest (10%, 17/177) consisted of a single, non-overlapping submovement. For instance, the example shown in Fig. 4C was considered to consist of a discrete, non-overlapping submovement, followed by a second, non-overlapping submovement with a peak velocity below our detection threshold (see Methods).

For the case of non-overlapping submovements, the average goodness of fit was 0.986±0.010 (N = 17) and 0.970±0.000 (N = 1) for flashing and stationary visual targets, respectively. For the case of 2 overlapping submovements, the goodness of fit was 0.989±0.008 (N = 126) and 0.990±0.007 (N = 149) for flashing and stationary visual targets, respectively. For the case of 3 overlapping submovements, the goodness of fit was 0.993±0.010 (N = 33) and 0.994±0.005 (N = 11) for flashing and stationary visual targets, respectively.

### Submovement parameters

**Figure 5:**
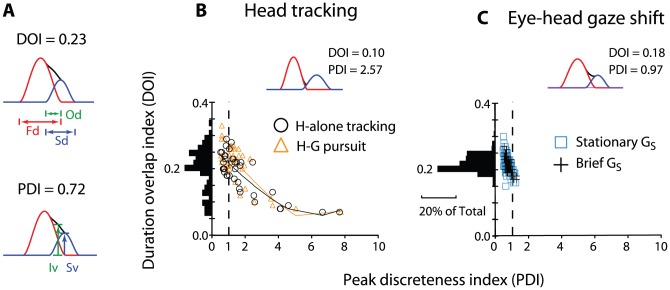
Two examples of the DOI and PDI measures and PDI vs. DOI scattergrams during head tracking movement and eye-head combined gaze shifts. The regression line across the data in **B** is a least-square function. The movement plotted in Fig. 3A is shown in the inset of **B**. Od: duration of submovement overlap; Fd: duration of the first submovement; Sd: duration of the second submovement; Iv: intercepted velocity between the first and second submovements: Sv: peak velocity of the second submovement. Data includes only overlapping submovements.

The degree of separation of the overlapping submovements was quantified by 2 measures, peak discreteness index (PDI) and duration overlap index (DOI; Fig. 5; see Methods). Figure 5A shows 2 examples of these measures (see Methods). The histogram for the head tracking DOI (Fig. 5B) clearly does not represent a normal distribution, as it is skewed towards higher values and shows relatively smaller peak at its mean value (0.20±0.06). By comparison, the eye-head gaze shift DOI histogram (Fig. 5C) is tightly clustered around its mean value (0.22±0.02). The DOI ranged from 0.1 to 0.6 ([0.1, 0.5] during head-alone tracking: [0.1, 0.6] during head-gaze pursuit; Fig. 5B). In contrast, during eye-head combined gaze shifts, the DOI ranged from 0.2 to 0.3 ([0.2, 0.3] for stationary target; [0.2, 0.3] for flashed target; Fig. 5B). The PDI measures also showed differences between the two conditions. Both the head tracking and eye-head gaze PDIs had the majority of their values clustered around a PDI of <1 (Fig. 5, B and C). However, the head tracking PDI graph displays a long tail with values extending up to 8. During head tracking, the PDI ranged from 0.3 to 7.8, (range: [0.3, 7.7] during head-alone tracking and [0.3, 7.8] during head-gaze pursuit), whereas during eye-head combined gaze shifts, the PDI ranged from 0.2 to 1.3 (range: [0.5, 1.2] for stationary target; range: [0.6, 1.3] for flashed target). The average PDI and DOI for head tracking was 1.7±1.5 (or logPDI = 0.13±0.28) and 0.20±0.06, respectively. The average PDI and DOI for eye-head combined gaze shifts was 0.8±0.1 (or logPDI = −0.12±0.07) and 0.22±0.02, respectively.

Note the average DOIs for head tracking and eye-head gaze shifts were very similar even though the ranges differed. However, the average PDI during head tracking was twice as high as that during eye-head combined gaze shifts. In addition, the standard deviation of the former was 15-fold higher than that of the latter. This suggests that the submovement overlapping was more stereotyped during eye-head combined gaze shifts than during head tracking. This finding was consistent with the measure of DOI, which indicated that the overlapping submovements were more distinct and separate during head tracking than during eye-head combined gaze shifts. This picture confirmed the earlier analysis, which showed that ∼60% (58–61%) of head tracking movements consisted of non-overlapping submovements, whereas <10% (1–10%) of the head movements accompanying gaze shifts did so.

**Figure 6:**
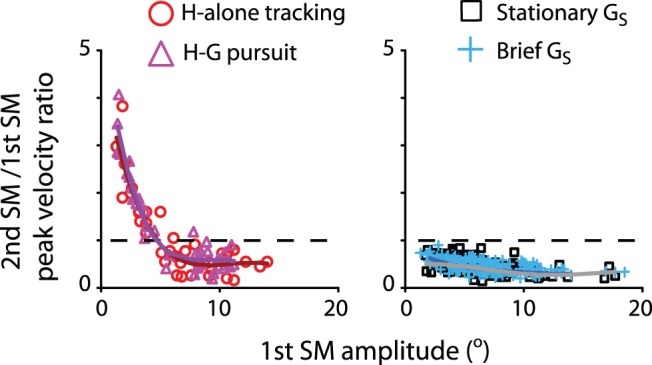
Head tracking movements and eye-head combined gaze shifts distinguished by the peak velocity ratio between the second and the first submovements as a function of submovement amplitude. The regression lines are least-square functions. The horizontal dashed lines indicate when the second submovements had the same values in peak velocity as the first submovement. Data includes only overlapping submovements.

The two-component strategy in goal-directed limb movements states that the initial submovement is always a larger component followed by relatively smaller, corrective submovements [16]–[18]. That is, the initial submovement should exhibit a relatively higher peak velocity than the second submovement. To assess this possibility, the peak velocity ratio between the second and first submovements is plotted in Fig. 6. For the head tracking movement (Fig. 6, left), this ratio decreased as a function of the first submovement amplitude, consistent with the need of head tracking. When the initial submovements were relatively small, it was more likely for the animals to recruit a second submovement with a relatively higher peak velocity, such that the animal could catch up with the moving target. In sharp contrast, this function is almost a flat line in the case of eye-head combined gaze shifts, suggesting that velocity compensation rarely occurred when the target was fixed in space (Fig. 6, right). This ratio was always lower than 1 (0.47±0.15 for a stationary target; 0.46±0.13 for a flashed target), indicating that the secondary submovement consistently exhibited a lower peak velocity than that of the initial submovement. That is, the secondary submovements served as a corrective movement, similar to the two-component strategy demonstrated in goal-directed limb movements [16]–[18].

**Figure 7:**
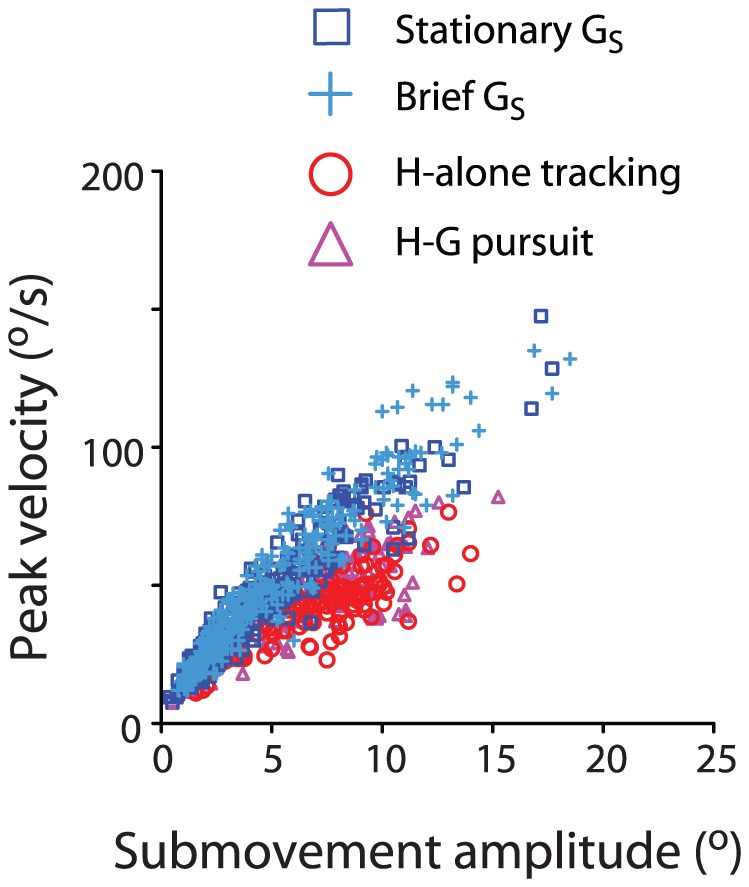
Head tracking movements and eye-head combined gaze shifts distinguished by peak velocity as a function of submovement amplitude. Data includes all submovements.

Figure 7 illustrates the relationship between peak velocity and submovement amplitude. Given that the amplitudes were the same (e.g. 10°), the head movement accompanying large eye-head combined gaze shifts consisted of submovements with relatively higher peak velocities compared to the head tracking movement (Fig. 7). There was a significant difference in this relationship (Heterogeneity-of-slope test, F = 37.46, p<.0001) between the head tracking movement (slope = 4.37, Pearson correlation r = .83) and the head movement accompanying gaze shifts (slope = 7.50, r = .96).

**Figure 8:**
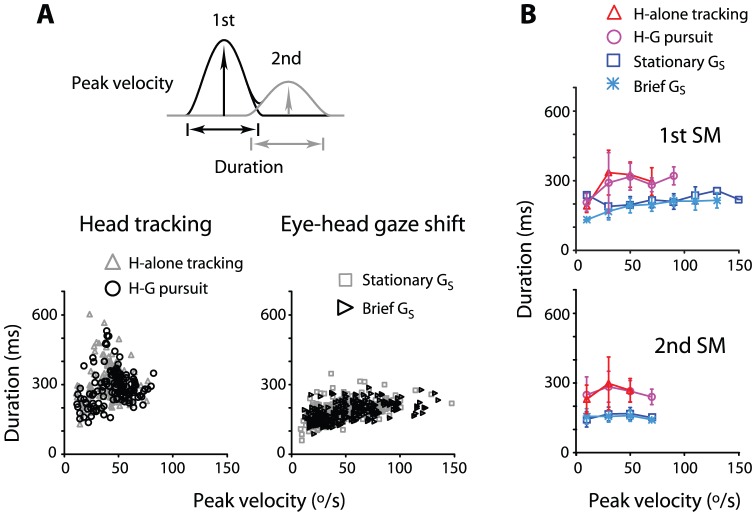
Head tracking movements and eye-head combined gaze shifts distinguished by submovement duration as a function of peak velocity. **A:** Data separated for head tracking and eye-head combined gaze shifts. **B**: Same data as in **A**, separated for first and second submovements. Data is plotted as mean ± S.D. Note during head tracking, the submovements exhibited relatively longer duration independent of peak velocity. This tendency persisted for the first and second submovements.

**Figure 9:**
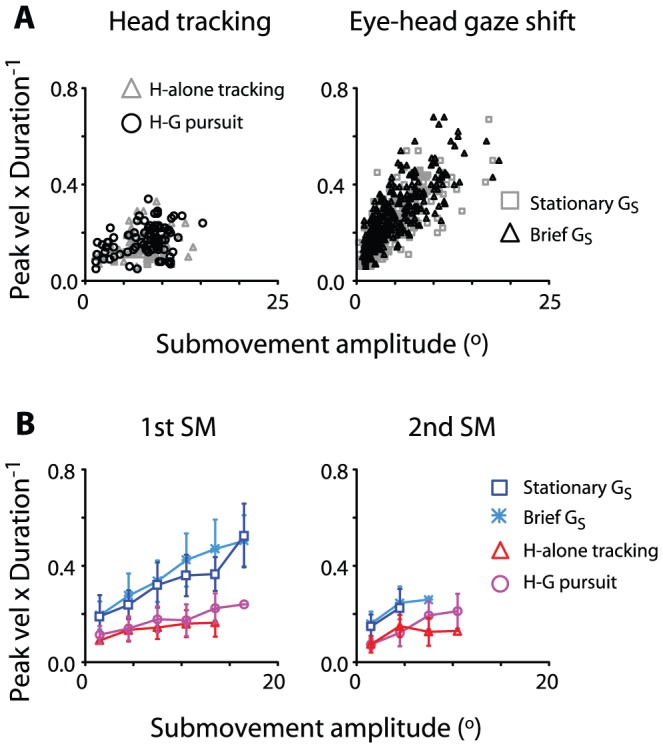
Head tracking movements and eye-head combined gaze shifts distinguished by normalized peak velocity (peak velocity×duration^−1^) as a function of submovement amplitude. Peak velocity and duration are computed after the example shown in Figure 6. Data is plotted as mean ± S.D. Note the submovements exhibited relatively higher normalized peak velocity during eye-head combined gaze shifts compared to head tracking movement. This tendency persisted independent of movement amplitude and the order of the submovements.

One parameter that distinguished the head movements from one another was submovement duration (Figs. 8 and 9). Figure 8 illustrates the submovement duration as a function of peak velocity. Head tracking movements had longer duration (320±73 ms and 297±85 ms for head tracking and head-gaze pursuit, respectively) than eye-head combined gaze shifts (202±40 ms and 195±37 ms for stationary target and flashed target, respectively; Fig. 8A). As shown in Fig 8B, differences in duration persisted across peak velocities regardless of the order of submovements (p<.01 except the data with <20°/sec for the first submovement; Fig. 8B).

The kinematic difference between head tracking movement and eye-head gaze shifts persisted even after submovement amplitude was considered. Figure 9A illustrates the normalized peak velocity, i.e., peak velocity/submovement duration, as a function of submovement amplitude. A clear difference in this measure was seen across amplitudes regardless of the order of submovements (p <.01 across all data points; Fig. 9B).

## Discussion

This study presents three major findings. First, the head movements observed during pursuit and gaze shifts were composed of a series of episodes, each consisting of distinct submovements that might or might not overlap with one another. The dynamics of the submovement could be accounted for by minimizing the rate of change of acceleration, or jerk, reminiscent of the previous findings in limb movement studies. Second, submovement composition varied according to the need for motor recruitment to perform the tasks. The head tracking movement often consisted of discrete, non-overlapping submovements, whereas the head movement accompanying rapid gaze shifts often consisted of submovements that overlapped one another. Third, the initial head submovement associated with eye-head combined gaze shifts had a higher peak velocity than that of the subsequent submovement, in agreement with the two-component strategy shown in goal-directed limb movements. Such an orderly relationship was absent during head tracking movements. These results extend the study of submovement composition from limb to head movements, suggesting that submovement composition provides a biologically meaningful approach to characterize these movements.

The question arises as to whether minimizing jerk is a biological constraint as opposed to a mathematical artifact. Our observations support the former possibility, in that *stand-alone* submovements with a bell-shaped velocity profile exist across all tasks, and the bell-shaped velocity profile was fit to a minimum-jerk model with the goodness of fit as high as 0.1% of error. This suggests that smooth, minimum-jerk head movement is a highly consistent observation [2]–[4]. Three additional lines of evidence indicated that submovement composition indeed reflects the motor recruitment required by task demand. First, the occurrence of non-overlapping submovements varied across task conditions. They were found frequently (∼60%) in head tracking movements, whereas they were a rarity (∼10%) in the head movements accompanying large gaze shifts. Second, the overlapping pattern among submovements varied from task to task. They exhibited a higher degree of separation from each other during head tracking movements than during eye-head combined gaze shifts (Fig. 5). Third, the relationship between peak velocity and submovement amplitude varied across tasks (Fig. 7), confirming that the kinematic characteristics of head movements were distinctly different between eye-head combined gaze shifts and head tracking (Figs. 8–9). These observations together suggest that the submovement composition of head movements reflects the motor recruitment by different task demands. This is consistent with past studies of other skeletomuscular movements [1]–[8], [11], [12], [16].

One may wonder why head movements are generated by a series of minimum-jerk submovements. In view of biomechanics, minimization of jerk may reduce “wear and tear” on the neuromuscular system [1], [3]. This protective design is needed for skeletomuscular movements across more than one joint [1], [24]. The smoothness may also improve predictability and hence control in the presence of sensing, communication, and actuation delays [5], [16], [18], [25]. In view of the flexibility of movement maneuvers, minimization of jerk provides a means to break up a given movement into sequences of scalable episodes, in which the sum of the episodes remains smooth [2], [5], [7]–[10]. The scalability feature of limb movement underlies the diversification of the movements [2]–[4]. The composition patterns of head submovements appeared highly diversified too. For instance, during eye-head combined gaze shifts, the primary submovement was sped up (i.e. duration shortened and peak velocity increased) in order to coordinate with saccadic eye movements and to achieve a rapid gaze shift. This was obviously not the case during head tracking. The tracking submovement was often slowed down (i.e. duration prolonged and peak velocity decreased) in order to match the motion of the juice spout (Figs. 8 and 9). This was because the target of gaze pursuit was different from the target of head tracking, and eye-head coordination was not needed to perform the task (see Methods). This shows that the submovement composition of the head movement is highly flexible, following the task demand, as opposed to a fixed pattern of eye-head movement.

It is of interest to note that the peak velocity of the primary submovement during eye-head combined gaze shifts was always higher than that of subsequent submovements (Fig. 6). The largest, primary head submovement was associated with orienting gaze shifts in the same direction; the secondary and/or tertiary submovement brought the head closer to its goal. In terms of timing, when the gaze shift was completed, the gaze command would have already ended. However, the head would continue moving, due to high inertia, while the head velocity descended to baseline. As a result, the primary head submovement would exhibit a velocity peak near gaze end) [26], cf. [27], for review, see [28]; Fig. 4). This temporal coupling with gaze end was absent in subsequent, smaller submovements, because there was no gaze shift. (Submovements recruited during and following a second, corrective gaze shift were excluded from our analyses; see Methods.) This suggests that the secondary submovement was recruited to correct the primary head submovement per se, *not* the gaze shift. One of the goals of the secondary (and tertiary) submovement might be maintaining the already stabilized gaze position while the head was in motion [28]–[32].

A similar pattern of magnitude order has been widely reported in goal-directed limb movements [4], [16]–[18], [25]. Woodworth (1899) proposed the two-component strategy model to account for the kinematics of the limb movement. The velocity profiles of these movements typically exhibit an “invariant” acceleration phase followed by a deceleration phase that varies from movement to movement [4], [16], [17]. He suggested that the initial, larger component is intended to bring the limb into the vicinity of the target. Once near the target, visual feedback provides fine adjustment to the movement trajectory in order to bring the limb to the target. Woodworth (1899) noted that the latter adjustments could take the form of “little extra movements” (p. 54) added after the initial limb movement [17]. In other words, the corrective adjustment is expressed as small, secondary submovement(s). Our observation for head movements agree with the general prediction of the two-component strategy model, suggesting that this strategy might be indeed common across the family of skeletomuscular movements. It is of interest that this two-component strategy is manifested in saccadic eye movements in a completely discrete manner, unlike its presentation in skeletomuscular movements. Saccades made to eccentric targets typically consist of an initial component, called primary saccade, which brings the line of sight close to the target. Following a refractory period of >100 ms, a so-called correction saccade follows, bringing the target to fovea. It is possible that the stereotyped eye-head coordinated relationship breaks down in patients who suffer from head movement disorders or strokes, similar to what has been shown in the limb movement deficits [11]–[13]. This possibility deserves to be explored further.

The two-component strategy was not always observed during head tracking (Fig. 6). The peak velocity of the initial submovement was not necessarily larger than that of subsequent submovements, not to mention that the submovements did not always overlap with one another. These head movements were aimed at tracking a juice spout in motion, as opposed to orienting toward a target fixed in space. From time to time, submovements of proper amplitudes were recruited in order to catch up with the juice spout's motion. The distinction of primary-vs.-corrective submovements became less clear-cut; hence, the secondary submovement did not necessarily exhibit a relatively lower peak velocity. This observation was consistent with that of past limb movement studies, in which subjects tracked or intercepted a moving target by limb movements [5], [8]. Even though these limb submovements substantially overlapped one another, their peak velocities could be higher than those of preceding submovements– unlike the two-component strategy seen in goal-directed limb movements [18]. These findings provided additional support to the notion that submovement composition reflects task-dependent patterns of motor recruitment [5], [8], [11]–[13], [16], [25].
